# Local regression transfer learning with applications to users’ psychological characteristics prediction

**DOI:** 10.1007/s40708-015-0017-z

**Published:** 2015-08-14

**Authors:** Zengda Guan, Ang Li, Tingshao Zhu

**Affiliations:** 1 Business School, Shandong Jianzhu University, Jinan, China; 2 Department of Psychology, Beijing Forestry University, Beijing, China; 3 Black Dog Institute, University of New South Wales, Sydney, Australia; 4 Institute of Psychology, Chinese Academy of Sciences, Beijing, China; 5 Institute of Computing Technology, Chinese Academy of Sciences, Beijing, China

**Keywords:** Local transfer learning, Covariate shift, Psychological characteristics prediction

## Abstract

It is important to acquire web users’ psychological characteristics. Recent studies have built computational models for predicting psychological characteristics by supervised learning. However, the generalization of built models might be limited due to the differences in distribution between the training and test dataset. To address this problem, we propose some local regression transfer learning methods. Specifically, k-nearest-neighbour and clustering reweighting methods are developed to estimate the importance of each training instance, and a weighted risk regression model is built for prediction. Adaptive parameter-setting method is also proposed to deal with the situation that the test dataset has no labels. We performed experiments on prediction of users’ personality and depression based on users of different genders or different districts, and the results demonstrated that the methods could improve the generalization capability of learning models.

## Introduction

In recent decades, people spend more and more time on Internet, which implies an increasingly important role of Internet in human lives. To improve online user experience, online services should be personalized and tailored to fit consumer preference. Psychological characteristics, including consistent traits (like personality [1]) and changeable status (like depression [2, 3]), are considered as key factors in determining personal preference. Therefore, it is critical to understand web user’s personal psychological characteristics.

Personal psychological characteristics can be reflected by behaviours. As one type of human behaviour, web behaviour is also associated with individual psychological characteristics [4]. With the help of information technology, web behaviours can be collected and analysed automatically and timely, which motivates us to identify web user’s psychological characteristics through web behaviours. Many studies have confirmed that it is possible to build computational models for predicting psychological characteristics based on web behaviours [5, 6].

Most studies build computational models by supervised learning, which learns computational models on labelled training dataset and then applies the models on another independent test dataset. Supervised learning assumes that the distribution of the training dataset should be identical to that of test dataset. However, the assumption might not be satisfied in many cases, e.g. demographic variation (e.g. variation of gender and district), which results in the low performance of trained models. Previous studies have paid little attention to this problem. In this paper, we build models based on an innovative approach, which do not need to make the assumption of identical distribution. Transfer learning, or known as covariate shift, is introduced and investigated for this purpose.

Most existing covariate shift methods compute the resampling weight of training dataset and then train a weighted risk model to predict on test dataset. Commonly, these researches use the entire dataset to reweight in the whole procedure. We notice that probability density of data points is similar to each other in their local neighbour region, and this motivates us to use only the local region instead of the whole dataset to improve prediction accuracy and save computation cost. Therefore, we bring in some local learning views to improve covariate shift. In addition, the situation can be encountered that people do not know any labels of the test dataset before they decide to predict them, so it is difficult to learn the parameters of learning model. To cope with this problem, we propose an adaptive parameter-setting method which needs no test dataset label. Besides, we focus on the regression form of local transfer learning since psychological characteristics labels are often used in the form of continual values.

In this paper, based on our previous work [7], we intend to work on more domains of psychological characteristics predictions and propose some new local regression transfer learning methods, including training-test* k*-NN method and adaptive* k*-NN methods, which are more effective and can adaptively set the unknown parameter in prediction functions.

The rest of the paper is organized as follows: we present the local regression transfer learning methods in Sect. 2; we then introduce the background of covariate shift and local learning, and propose some local transfer learning methods to reweight the training dataset and build the weighted risk regression model. We perform some experiments of psychological characteristics prediction and analyse the experiment results in Sect. 3. Finally, we conclude the whole work in the last section.

## Local regression transfer learning

### Covariate shift

In this paper, the input dataset is denoted by *X* and its labels are denoted by *Y*. The training dataset is defined as $Z_{\text{tr}}=\{(x^{(1)}_{\text{tr}}, y^{(1)}_{\text{tr}}),...,(x^{(n_{\text{tr}})}_{\text{tr}}, y^{(n_{\text{tr}})}_{\text{tr}})\}\subseteq {\mathcal{X}} \times {\mathcal{Y}}$ with a probability distribution $P_{\text{tr}}(X,Y)$, and the test dataset is defined as $Z_{\text{te}}=\{(x^{(1)}_{\text{te}}, y^{(1)}_{\text{te}}),...,(x^{(n_{\text{te}})}_{te}, y^{(n_{\text{te}})}_{\text{te}})\}\subseteq {\mathcal{X}}\times {\mathcal{Y}}$ with a probability distribution $P_{\text{te}}(X,Y)$.

It is quite often that the test dataset has a different distribution from the training dataset. We focus on simple covariate shift that only inputs of the training dataset and inputs of the test dataset follow different distributions, i.e. only $P_{\text{tr}}(X)\ne P_{\text{te}}(X)$, while anything else does not change [8].

Then, we will introduce a general solution framework to cope with covariate shift problems. The key point is to compute probability of training data instances within the test dataset population, so that people can use labels of the training dataset to learn a test dataset model. We illustrate the process as [9, 10] did.

Firstly, we represent the risk function in this situation and minimize its expected risk:

1 $$\begin{aligned} \min _\theta E_{(x_{\text{tr}},y_{\text{tr}}) \sim P_{\text{te}}} l(x_{\text{tr}},y_{\text{tr}},\theta ) \end{aligned},$$

where $l(x_{\rm tr},y_{\rm tr},\theta )$ is the loss function, which depends on an unknown parameter $\theta$, and $(x_{\rm tr},y_{\rm tr})\sim P_\text{te}$ denotes the probability with which $(x_{\rm tr},y_{\rm tr})$ belongs to test dataset population.

It is usually difficult to compute the distribution of $P_{\text{te}}$, so people turn to compute the empirical risk form as follows:

2 $$\begin{aligned} \min _\theta E_ {(x,y)\sim P_{\rm tr}}\frac{P_\text{te}(x_{\rm tr}, y_{\rm tr})}{P_{\rm tr}(x_{\rm tr},y_{\rm tr})}l(x_{\rm tr},y_{\rm tr},\theta )\nonumber \\ \approx \min _\theta \frac{1}{n_{\rm tr}}\sum _{i=1}^{n_{\rm tr}} \frac{P_{\text{te}}(x_{\rm tr},y_{\rm tr})}{P_{\rm tr}(x_{\rm tr},y_{\rm tr})} l(x_{\rm tr},y_{\rm tr},\theta ). \end{aligned}$$

It is usually assumed that $P_{\text{tr}}(y|x)=P_{\text{te}}(y|x)$, i.e. the prediction functions for both datasets are identical. Then, $\frac{P_{\text{te}}(x_{\text{tr}},y_{\text{tr}})}{P_{\text{tr}}(x_{\text{tr}},y_{\text{tr}})}$ is replaced by $\frac{P_{\text{te}}(x_{\text{tr}})}{P_{\text{tr}}(x_{\text{tr}})}$. People usually directly compute the ratio $\frac{P_{\text{te}}(x_{\text{tr}})}{P_{\text{tr}}(x_{\text{tr}})}$ but do not estimate $P_{\text{tr}}$ and $P_{\text{te}}$ independently, which can avoid generating more errors.

To estimate the ratio $\frac{P_{\text{te}}(x_{\text{tr}})}{P_{\text{tr}}(x_{\text{tr}})}$ , also called the importance, researchers construct many kinds of forms of formula 2. Sugiyama et al. [11] computed the importance by minimizing the Kullback–Leibler divergence between training and test input densities and constructed the prediction model with a series of Gaussian kernel basis functions. Kanamori et al. [12] proposed a method which minimizes squares importance biases represented by Gaussian kernel functions centred at test points. Huang et al. [10] used a kernel mean matching method (KMM) which computed the importance by matching test and training distributions in a reproducing-kernel Hilbert space. Dai et al. [13] and Pardoe et al. [14] proposed a list of boosting-based algorithms for transfer learning.

### Local machine learning

Local machine learning has shown a comparative advantage in many machine learning tasks [15–17]. In some situations, the size of local region of target data imposes a significant effect on prediction accuracy of model [17]. On the one hand, too many neighbour points can over-estimate the effects of long-distance points which may have little relationship with target point. Thus, this may bring unnecessary interferences to learning process and produce more computation cost. In another way, the predicted data point can be thought to have similar property only to points in its small region but not to all points in a very big region. On the other hand, too less neighbour points may introduce strong noise to local learning.

For covariate shift, density estimation is important. There are many density estimation methods including k-nearest-neighbour methods, histogram methods and kernel methods, which are localized with only a small proportion of all points which contribute most to the density estimation of a given point [18]. The k-nearest-neighbour approximation method is represented as follows:

3 $$\begin{aligned} P(x) = \frac{k}{nV} \end{aligned},$$

where *k* is the number of nearest neighbours, *n* is the total number of all data and *V* is the region volume containing all nearest neighbours. If the training and test data are in one volume, ratio between densities of both can be represented as $k_{\text{tr}}/k_{\text{te}}$, which do not require to compute *nV* any more. Moreover, Loog [19] proposed a local classification method which estimated the importance by using the number of test data falling in its neighbour region which consisted of training and test data. All of these inspired us to further study local learning within covariate shift.

### Reweighting the importance

A complete covariate shift process is divided into two stages: reweighting importance of training data, and training a weighted machine learning model for prediction on the test dataset. In the first stage, we reweight the importance of training instances by estimating the ratio $P_{\text{te}}(x_{\text{tr}})/P_{\text{tr}}(x_{\text{tr}})$.

In this work, we use local learning to improve the performance in covariate shift. The key point is to use the neighbourhood of training points to compute their importance. In fact, this uses the knowledge of density similarity between the training point and its neighbour points. K-nearest-neighbour and clustering methods are used to determine the neighbourhood of training point and reweight the importance. Specifically, we first present * k*-NN reweighting method, which is simplest and can be seen as an origin form of all our* k*-NN methods. Training-test* K*-NN reweighting method is an extension of* k*-NN reweighting method, and adaptive* K*-NN reweighting method is an adaptation of training-test* K*-NN reweighting method to more common situations. Clustering-based reweighting method is another view about using local learning to reweight the importance.

#### *K*-NN reweighting method

We firstly introduce k-nearest-neighbour reweighting methods [7], which uses *k*-nearest test set neighbours of training instance to compute its importance. Gaussian kernel is chosen to compute density distance between training data and test data. Then the importance can be computed as follows:

4 $$\begin{aligned} {\text{Weig}}(x_{\text{tr}}) = \sum _{i=1}^{k} {\text{exp}}\left(-\gamma ||x_{\text{tr}} - x^{(i)}_{\text{te}}||^2_2\right) \end{aligned},$$

where *k* represents the number of the nearest test set neighbours of training data $x_{\text{tr}}$, which determines the size of the local region, and * γ* reflects the bandwidth of kernel function and $\gamma > 0$. Even though the exponential term in ${\text{Weig}}(x_{\text{tr}})$ decreases according to an exponential law, the *k* value is helpful for obtaining an appropriate neighbour region and then computing the importance. It is easy to know that this k-nearest-neighbour reweighting method can save much computation time when the size of dataset is very large compared with *k*.

#### Training-test* K*-NN reweighting method

When we regard both the training and test neighbours of given training data in a local region, we develop a new k-nearest-neighbour reweighting method, called training-test* k*-NN reweighting method, which uses both training data and test data. The training-test * k*-NN reweighting method tries to use more training data points to balance the effect which is due to that the only training point does not have comparable probability with the other test points in the * k*-NN reweighting method sometimes, which may reduce the performance of the * k*-NN method. Simply, $k_{\text{tr}}/k_{\text{te}}$ can be used as a reweighting formula if the training data and test data in the local region are treated to have similar probability. Further, we put forward the below formula to compute the importance after combining Gaussian kernels.

5 $$\begin{aligned} {\text{Weig}}(x_{\text{tr}}) = \frac{\frac{1}{k_{\text{te}}}\sum _{i=1}^{k_{\text{te}}} {\text{exp}}(-\gamma ||x_{\text{tr}} - x^{(i)}_{\text{te}}||^2_2)}{\frac{1}{k_{\text{tr}}}\sum _{j=1}^{k_{\text{tr}}} {\text{exp}}(-\gamma ||x_{\text{tr}} - x^{(j)}_{\text{tr}}||^2_2)} \end{aligned},$$

where the neighbour region divides into two parts: the training data part with a total number of $k_{\text{tr}}$ and the test data part with a total number of $k_{\text{te}}$. The total number of data in the neighbour region is $k = k_{\text{tr}} + k_{\text{te}}$. When we determine the *k*, $k_{\text{tr}}$ and $k_{\text{te}}$ will be determined automatically. Here, since the training point itself is also defined as its neighbour, the denominator cannot be 0.

#### Adaptive* K*-NN reweighting method

For covariate shift methods, how to determine appropriate parameters is an important issue. Cross validation technique is used broadly for the problem. However, cross validation technique needs some labelled test data to be as validation dataset. When the prediction model is used in changed situation where test data are completely not labelled, people cannot apply cross validation. Here, we give an empirical parameter estimation way to modify the training-test* k*-NN reweighting method. We call it adaptive* k*-NN reweighting method, which includes how to determine *k* and how to determine * γ*.

For *k*, we first assign $k\approx n^\frac{3}{8}$ in the way of Enas and Choi [20], where *n* is the population size. Then we reduce *k* to be a smaller value $n_{\text{neig}}$ when Gaussian kernel function ratio $gau(n_{\text{neig}}+1)/gau(n_{\text{neig}})$ is less than a threshold, which makes data in the region have similar probability. *gau*(*i*) is defined as ${\text{exp}}(-\gamma ||x_{tar} - x^{(i)}||^2_2)$. The reason is that, if a too small value *gau*(*i*) of nearest-neighbour point *i* is summed to compute the density together with other big values, that would bring big bias, and thus the point should be gotten rid of.

As to the parameter* γ*, we set it as an empirical way $\gamma = \frac{1}{2n_{\text{neig}}}\sum _{i=1}^{n_{\text{neig}}} ||x_{\text{tr}} - x^{(i)}||^2_2)$. In fact, this way is somehow like a way of computing an approximated empirical variance of a dataset.

#### Clustering-based reweighting method

Finally, we introduce clustering-based reweighting methods [7], which are somehow similar to data-adaptive histogram method [18]. This kind of methods use clustering algorithm to generate histograms, whereas it uses training and test instances in one histogram to estimate the importance. In detail, clustering is performed on the whole training and test dataset, and $P_{\text{te}}(x_{\text{tr}})/P_{\text{tr}}(x_{\text{tr}})$ is estimated through computing the ratio between number of test data and number of training data in one cluster. The idea is simple that training data and test data clustered in one small enough region can be thought to have the equal probability and then the importance can be computed with the ratio. Thus, we obtain the formula of clustering-based reweighting method as follows:

6 $$\begin{aligned} {\text{Weig}}(x_{\text{tr}}^{(i)}) =\frac{|{\text{Clus}}_{\text{te}}(x_{\text{tr}}^{(i)})|}{|{\text{Clus}}_{\text{tr}}(x_{\text{tr}}^{(i)})|} \end{aligned},$$

where ${\text{Weig}}(x_{\text{tr}}^{(i)})$ denotes the importance of training data $x_{\text{tr}}^{(i)}$, and $|{\text{Clus}}_{\text{tr}}(x_{\text{tr}}^{(i)})|$ and $|{\text{Clus}}_{\text{te}}(x_{\text{tr}}^{(i)})|$ denote, respectively, the number of training data and the number of test data in the same cluster which contains $x_{\text{tr}}^{(i)}$.

Like the histogram method, this method may suffer from high-dimensional difficulty. Number of training data and test data in their cluster affects the probability estimation, and it needs very many data in high-dimensional situation. Clustering method also has a big influence on risk of importance weighting, because common clustering methods are not accurate density-region division methods. Clustering-based reweighting method can be taken as an approximate computation way.

### Weighted regression model

When we get the importance of all training data in the previous stage, we train the weighted learning model and predict on the test dataset. The importance of training data is taken as weight of data and is integrated into the following formula:

7 $$\begin{aligned} \min \sum _{i=1}^{n_{\text{tr}}} {\text{Weig}}\left(x^{(i)}_{\text{tr}}\right) \cdot l\left(y^{(i)}_{\text{tr}}, f\left(x^{(i)}_{\text{tr}}\right)\right) \end{aligned},$$

where ${\text{Weig}}(x^{(i)}_{\text{tr}})$ denotes the importance of training instances $x^{(i)}_{\text{tr}}$ and $l(y^{(i)}_{\text{tr}},\, f(x^{(i)}_{\text{tr}}))$ represents the bias between the real value $y^{(i)}_{\text{tr}}$ and the prediction value $f(x^{(i)}_{\text{tr}})$ which is a regression function. It can be seen that each instance in the weighted model has a different weight, while the weight in unweighted models is uniform.

In this work, we integrate multivariate adaptive regression splines (MARS) method with local reweighting methods. MARS is an adaptive stepwise regression method [21], and its weighted learning model has the following form:

8 $$\begin{aligned}&\min \sum _{i=1}^{n_{\rm tr}} {\text{Weig}}\left(x^{(i)}_{\rm tr}\right) \cdot \left(y^{(i)}_{\rm tr} - f\left(x^{(i)}_{\rm tr}\right)\right)^2\nonumber \\&f(x^{(i)}_{\rm tr}) = \beta _0 + \sum _{j=1}^{m} \beta _j h_j\left(x^{(i)}_{\rm tr}\right), \end{aligned}$$

where $h_j(x)$ is a constant denoted by *C*, or a hinge function with the form ${\text{max}}(0, x-C)$ or ${\text{max}}(0, C-x)$, or a product of two or more hinge functions. *m* denotes the total steps to get optimal performance, and $f(x^{(i)}_{\text{tr}})$ and $f(x^{(i)}_{\text{te}})$ denote the prediction values of training data and test data, respectively. This model is trained for solving unknown coefficients $\beta _j$.

## Experiments

Our experiments aim to predict microblog users’ psychological characteristics. They include three parts: predicting users’ personality across different genders, predicting users’ personality across different districts and predicting users’ depression across different genders.

In this paper, personality is evaluated by the Big Five personality framework, a wide accepted personality model in psychology. The Big Five personality model describes human personality with five dimensions as follows: agreeableness (A), conscientiousness (C), extraversion (E), neuroticism (N) and openness (O) [22]. Agreeableness refers to a tendency to be compassionate and cooperative. Conscientiousness refers to a tendency to be organized and dependable. Extraversion refers to a tendency to be socialized and talkative. Neuroticism refers to a tendency to experience unpleasant emotions easily. Openness refers to the degree of intellectual curiosity, creativity and a preference for novelty. Besides, CES-T scale [23] is employed to measure web users’ depression.

We test the local transfer methods among web users with different genders and in different districts. There exists some relationship between users’ web behaviours and their personality/depression. Gender is an important factor that can effect users’ behaviours, so we choose it as example to test the local transfer methods. It is often encountered that users of the training set and the test set are in different districts, so we also study the suitability of the local transfer methods in this situation. Depression in male and female shows difference [24], so we also investigate it. In detail, our experiments are to predict male users’ personality based on female users, predict non-Guangdong users’ personality based on Guangdong users and predict male users’ depression degree based on female users.

### Experiment setup

In China, Sina Weibo (weibo.com) is one of the most famous microblog service providers and has more than 503 million registered users. In this research, we invited Weibo users to complete online self-report questionnaire, including personality and depression scales, and downloaded their digital records of online behaviours with their consent.

For the prediction of personality, between May and August in 2012, we collected data from 562 participants (male: 215, female: 347; Guangdong: 175, non-Guangdong: 387) and extracted 845 features from their online behavioural data. The extracted features can be divided into five categories: (a) profiles include features like registration time and demographics (e.g. gender); (b) self-expression behaviours include features reflecting the online expression of one’s personal image (e.g. screen name, facial picture and self-statement on personal page); (c) privacy settings include features indicating the concern about individual privacy online (e.g. filtering out private messages and comments sent by strangers); (d) interpersonal behaviours include features indicating the outcomes of social interaction between different users (e.g. number of friends whom a user follows, number of followers, categories of friends whom a user follows and categories of forwarded microblogs); and (e) dynamic features can be represented as time series data (e.g. updating microblogs in a certain period or using apps in a certain period).

For the prediction of depression, between May and June in 2013, we collected data from 1000 participants (male: 426, female: 574). Compared with personality experiments, we supplemented additional linguistic features in depression experiments. These linguistic features included the total number of characters, the number of numerals, the number of punctuation marks, the number of personal pronouns, the number of sentiment words, the number of cognitive words, the number of perceptual processing words and so on.

Since all these experiments have very many feature dimensions and high dimension curse would weaken the learning model, we firstly use stepwisefit method in Matlab toolbox to reduce dimensions and select the most relevant features. For the gender-personality experiment, we process the female dataset and obtain 25, 14, 19, 25 and 20 features for predicting Big Five dimensions: A, C, E, N and O, respectively. For the district-personality experiment, the Guangdong dataset is processed and we obtain 19, 21, 18, 22 and 20 features for A, C, E, N and O, respectively. For the depression experiment, the female dataset is processed, and we obtain 20 features.

It also must be emphasized that we test whether the training set and the test set follow the same distribution before we do transfer learning. Both *T* test and Kolmogorov–Smirnov test are performed in the two-sample test.* T* test is fit to test dataset with Gaussian distribution, and Kolmogorov–Smirnov test can test dataset with unknown distribution. Specifically, we test the datasets along each dimension.

In the experiments, our local transfer learning methods are compared with non-transfer method, global transfer method and other transfer learning methods. The local transfer learning methods include* k*-NN transfer learning method, training-test* k*-NN transfer learning method, adaptive* k*-NN transfer learning methods and clustering transfer learning methods. The non-transfer method does not use a transfer learning way and is a traditional method. The global transfer method is also a* k*-NN transfer learning method, but it has a *k* value equalling the number of all test data, i.e. it takes all test data as neighbours. A famous transfer learning method called KMM [10] is also used here as a baseline method. After reweighting importance, we integrate the importance into weighted risk models. We choose weighted risk model MARS, which is open source regression software for Matlab/Octave from (http://www.cs.rtu.lv/jekabsons/regression.html).

In all tables and figures of this paper, MARS denotes the method with no transfer learning, KMM denotes combination of KMM reweighting method and MARS method in a weighted risk form, GkNN denotes global* k*-NN reweighting method and MARS, kNN denotes* k*-NN reweighting method and MARS, TTkNN denotes training-test* k*-NN reweighting method and MARS, and AkNN1 denotes adaptive* k*-NN reweighting method and MARS, where *k* value is determined as described in Sect. 2.3.3. AkNN2 denotes completely adaptive* k*-NN reweighting method and MARS, where *k* value and* γ* value are both determined as described in Sect. 2.3.3. Clust denotes clustering-based reweighting method and MARS. KMM, GkNN, kNN, TTkNN, AkNN1 and Clust all showed the best results where their parameter values are assigned the best of a series of tried values. In all experiments, we use mean square error (MSE) for result comparisons.

### Predicting users’ personality across genders

This task is to predict male users’ personality based on female users’ labelled data and male users’ unlabelled data. We firstly perform single-dimension* T* test and Kolmogorov–Smirnov test to test whether male and female datasets are drawn from the same distribution. As a result, 3, 1, 2, 3 and 2 features of all 25, 14, 19, 25 and 20 features are shown to follow different distributions by* T* test, and 2, 0, 0, 2 and 1 features by Kolmogorov–Smirnov test. All of these test results are with probability more than 95 % confidence. Thus, it can be thought that there exists some distribution divergence between male and female datasets, though the divergence is not big. Then, we examine the performance of all the local transfer learning methods in this experiment.

**Table 1:** Local regression transfer learning results for predicting personality across different-gender datasets. MSE is used to measure the test results

Condition	A	C	E	N	O
MARS	34.8431	45.9335	34.0655	29.5776	32.6700
KMM	26.7654	30.8683	24.0116	27.9208	28.1425
GkNN	25.2125	31.5119	23.1247	27.6345	30.6127
kNN	24.3776	31.1357	23.1247	27.4160	28.2948
TTkNN	24.3149	31.0282	22.8547	27.8493	28.1424
AkNN1	24.3913	31.2013	24.5649	27.4419	28.2027
AkNN2	29.8956	31.0112	24.0063	27.8779	28.1899
Clust	27.3070	30.4555	23.9003	27.7718	28.1425

From Table 1, it can be seen that all regression transfer learning methods improve much on the prediction accuracy compared with non-transfer learning method in all situations. Local kNN reweighting methods beat global k-NN reweighting method GkNN in almost all situations. TTkNN method performs better than the others in 3 of 5 personality dimensions. AkNN1 performs nearly well with other* k*-NN reweighting methods, except in the dimension of C. Especially, AkNN1 beats GkNN in 4 dimensions, and this shows the advantage of its fixed *k* value. For AkNN2, it performs better only than MARS method. Clust also shows comparable performance compared with other local transfer learning methods.

**Fig. 1:**
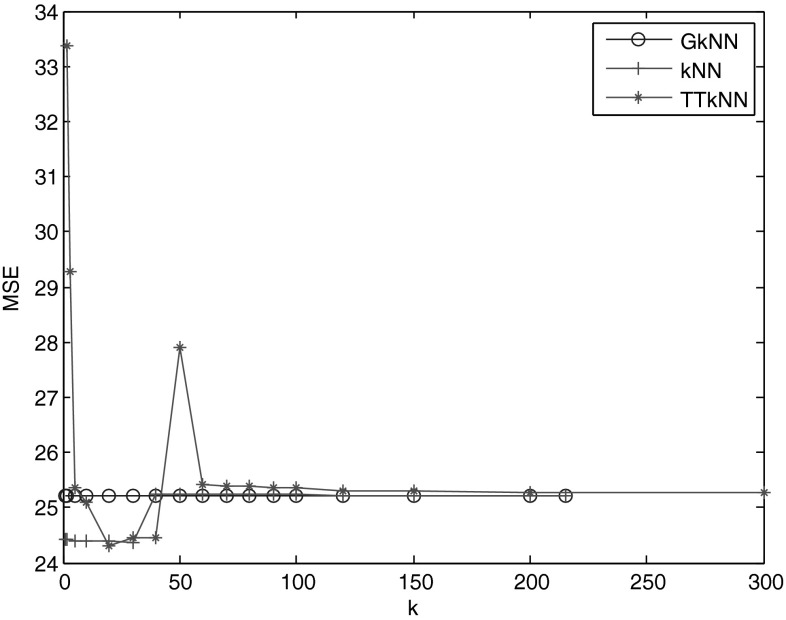
The impact of the number of nearest neighbours on the performance of* k*-NN transfer methods in trait A

To investigate the impact of *k* value in* k*-NN reweighting methods, we take experiment on trait A as an example. The results of GkNN, kNN and TTkNN are shown in Fig. 1. We can see that these methods perform the best when the values of *k* range between 20 and 30. As *k* approximates to the total size of test dataset, the performances of kNN and TTkNN become equal to GkNN method. For TTkNN method, it performs worse than GkNN when *k* is 1, and that could be caused by noise. When *k* of TTkNN method is very small, i.e. close to 0, outlier point can impose a strong influence. When *k* of TTkNN method is 50, its performance shows an exception and the reason may be that the local region caused by *k* experiences a shake-up. Thus, the value of *k* can be recognized as a factor affecting the prediction performance.

**Fig. 2:**
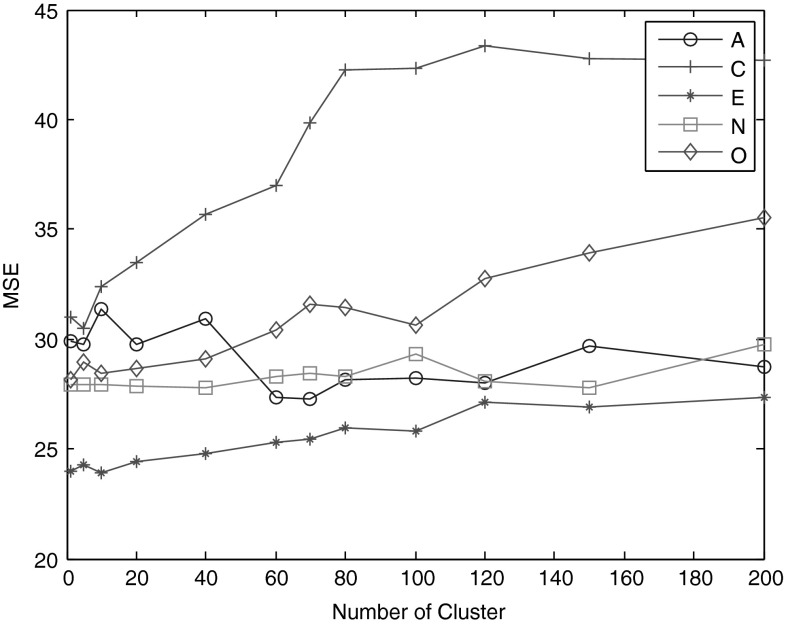
The impact of cluster number in clustering regression transfer learning in trait A, C, E, N and O

We then test how prediction accuracy of clustering transfer methods is affected by the number of clusters in all five personality traits. From Fig. 2, we can see that the number of clusters has a big influence on the prediction accuracy. There is no certain value of cluster number which achieves the best performance for all five traits. The method obtains the optimization result in C, E and O trait when the number of clusters is small. For these three traits, it could also be seen that their MSE gradually increases as number of clusters increases, and the least *k* value (here, the value is 1) may not be the optimised value because of noise. Meanwhile, it seems to follow no regular rule for the other two traits. Thus, we can think that there is no constant optimal value for cluster number in clustering transfer methods for all situations. The reasons are speculated that distributions of the datasets are of diversity, and clustering method is not a stable density estimation method here.

### Predicting users’ personality across districts

In this experiment, we use Weibo data of Guangdong province of China to train the model and predict personality of users in the other districts. Firstly, we still apply stepwisefit method to select 19, 21, 18, 22 and 20 features from a total of 845 features in A, C, E, N and O traits, respectively. We then use* T* test and get 3, 1, 3, 3 and 2 features following different distributions and use Kolmogorov–Smirnov test and get 3, 5, 6, 9 and 2 features following different distributions, both with probability more than 95% confidence. Finally, we perform our regression transfer methods on different-district datasets and compare all the methods as used in the above different-gender experiment.

**Table 2:** Local regression transfer learning results for predicting personality across different-district datasets. MSE is used to measure the test results

Condition	A	C	E	N	O
MARS	43.6764	65.0172	44.3688	47.4115	229.8742
KMM	42.1194	48.9055	39.3781	47.4057	59.7330
GkNN	44.7136	45.8609	43.0928	49.2114	43.1696
kNN	43.2840	42.0574	38.8104	42.9135	43.1696
TTkNN	38.6335	40.8370	35.0338	41.8510	45.3623
AkNN1	43.5722	41.3360	39.0398	41.4173	195.6540
AkNN2	41.5186	62.8834	39.3683	52.2294	218.9917
Clust	39.1079	42.5235	37.7979	44.6171	113.6659

We analyse performances of all methods. Table 2 shows that all local transfer learning methods perform better than non-transfer method MARS. GkNN behaves unstably: it performs worse than MARS in 2 of all 5 traits, while it performs best in O trait. kNN performs no worse than GkNN in all five traits. TTkNN is still the best method for most situations and performs stably. AkNN1 performs much better than MARS, but much worse in O trait than other local transfer learning methods except AkNN2. AkNN2 behaves only a little better than MARS in four traits and weaker in one trait. Clust also beats MARS method in all situations but behaves not so well in O trait.

### Predicting users’ depression across genders

This experiment is to predict male users’ depression level based on female users’ labelled data. Still, stepwisefit method is performed and 20 features are selected. 3 feature dimensions in* T* test and 5 feature dimensions in Kolmogorov–Smirnov test are thought as different-distribution feature. This suggests that training and test data also follow different distributions in this experiment.

**Table 3:** Local regression transfer learning results for predicting depression across different-gender dataset

	MARS	KMM	GkNN	kNN	TTkNN	AkNN1	AkNN2	Clust
MSE	126.9868	111.2089	113.5784	113.4111	113.1296	113.3482	113.3624	111.5430

In Table 3, the result shows that the transfer learning methods perform much better than non-transfer method MARS. KMM and Clust behave a little better than other transfer methods. AkNN1 and AkNN2 perform nearly equally well to other transfer learning methods.

### Discussion and conclusion

It can be concluded from the above experiments that all our local transfer learning methods work better than non-transfer learning method, because they reduce the prediction bias of model which is trained and tested on different-distribution datasets. Our local* k*-NN family transfer learning methods perform better than the global* k*-NN transfer learning method generally, and the reason may be that an appropriate *k* value in* k*-NN methods could reflect more subtle nature in density estimation. All our local transfer learning methods show comparable performance with KMM method in all situations. TTkNN method exceeds kNN and obtains the best performance among all the methods in half of situations. It could be guessed that TTkNN uses both test and training data information, while kNN only uses test data. Clust method performs well in most situations, this proves its applicability, and better density clustering methods may further enhance this method.

Finally, we compare the performance of GkNN, AkNN1 and AkNN2; AkNN1 is the best, GkNN is the second and AkNN2 is the worst of them. AkNN1 performs better than GkNN in most situations, and this demonstrates that determining *k* in an AkNN1 way, same as AkNN2, can work well generally. We also note that AkNN1 and AkNN2 behave not well in O trait in Table 2, and it indicates that *k* in AkNN1 and AkNN2 is not an optimal choice in some situation because of the change of distribution of data set. It is pointed that AkNN2 is inferior to AkNN1 and GkNN, because it does not choose the optimal value for parameter* γ* in prediction function preliminary. Since no parameter in AkNN2 needs to be set artificially, it could work in the situations where we completely have no idea about labels of predicted data, which can be of much significance.

## Conclusions

In this paper, we propose some local regression transfer learning methods and apply them to predict users’ psychological characteristics when the training set and the test set follow different distributions. We present* k*-NN reweighting methods and clustering reweighting method to estimate the importance of training set in covariate shift process. Specifically, these methods utilize training and test data in certain local neighbour region for importance estimations. We still apply them to psychological characteristics predictions including microblog users’ personality prediction across different genders and different districts, and microblog users’ depression prediction across different genders. The experiments demonstrate that these methods improve the accuracy of prediction models. Specially, the complete adaptive* k*-NN reweighting method is able to make prediction even without knowing any label of test data.
